# Alveolar and Bronchial Nitric Oxide Parameters in Pre-Capillary Pulmonary Hypertension

**DOI:** 10.3390/biomedicines13122957

**Published:** 2025-12-01

**Authors:** Balázs Csoma, Gergő Szűcs, András Bikov, Zsolt Dezső Rozgonyi, Alexandra Nagy, Zsombor Matics, Veronika Müller, Kristóf Karlócai, Györgyi Csósza, Zsófia Lázár

**Affiliations:** 1 Department of Pulmonology, Semmelweis University, 1083 Budapest, Hungary; csoma.balazs@semmelweis.hu (B.C.); szucs.gergo@semmelweis.hu (G.S.); nagy.alexandra@semmelweis.hu (A.N.); matics.zsombor@semmelweis.hu (Z.M.); karlocai.kristof@semmelweis.hu (K.K.); csosza.gyorgyi@semmelweis.hu (G.C.); 2 Wythenshawe Hospital, Manchester University NHS Foundation Trust, Manchester Academic Health Science Centre, Manchester M23 9LT, UK; andras.bikov@gmail.com; 3 Division of Infection, Immunity & Respiratory Medicine, Faculty of Biology, Medicine and Health, The University of Manchester, Manchester M23 9LT, UK; 4 Department of Anesthesiology and Intensive Therapy, Semmelweis University, 1083 Budapest, Hungary; rozgonyi.zsolt.dezso@semmelweis.hu

**Keywords:** exhaled nitric oxide, extended nitric oxide analysis, pulmonary hypertension, lung, alveolar inflammation, exercise capacity

## Abstract

**Background:** Exhaled NO concentrations at different flow rates can be used to calculate pulmonary NO dynamics in the conductive and peripheral airways and can be described by the total bronchial flux of NO (*J*awNO) and alveolar NO concentration (*C*ANO), respectively. Changes in these parameters have been shown in pre-capillary pulmonary hypertension (PH); however, data from studies with low sample sizes are controversial and did not prospectively assess *J*awNO and *C*ANO after adequate therapy. **Methods:** Patients with untreated pre-capillary PH (group 1: N = 23, group 3: N = 11, group 4: N = 18) and control subjects (N = 27) were recruited in a single-center observational study. Patients with group 1 (N = 15) and group 4 PH (N = 13) also attended a single follow-up visit when on pulmonary vasodilators or following interventions. Exhaled NO concentrations were measured at 50 mL/s and 100–250 mL/s expiratory flows and the two-compartment linear model was used for the calculation of *J*awNO and *C*ANO. **Results:** *C*ANO was higher in patients (median (interquartile range) 3.84 (2.64–7.29) ppb) than in control subjects (2.70 (1.85–4.29) ppb, *p* < 0.01; Mann–Whitney test) without a difference among PH groups or an association with survival. *C*ANO showed moderate negative associations with the diffusion capacity of the lung for carbon monoxide (Spearman r = −0.41, *p* < 0.01) and a trend for mortality risk categories in groups 1 and 4 (r = −0.30, *p* = 0.06). Only *J*awNO changed at follow-up (0.69 (0.14–1.10) vs. 0.91 (0.40–1.68) nL/s, *p* = 0.02; Wilcoxon test), and there was a positive correlation between its increase and the improvement in 6 min walk distance (r = 0.40, *p* = 0.04). **Conclusions:** Alveolar NO concentration is increased in patients with pre-capillary PH, and the change in *J*awNO is related to the improvement in exercise capacity in PH groups 1 and 4. This is the first study implying that *J*awNO might be a non-invasive marker responsive to improved pulmonary hemodynamics in PH.

## 1. Introduction

Pre-capillary pulmonary hypertension (PH) encompasses diseases of multiple origins, including pulmonary arterial hypertension (PAH, group 1), PH due to lung diseases and/or hypoxia (group 3), pulmonary artery obstruction (group 4), and PH with unclear or multifactorial mechanisms (group 5) [1]. Pulmonary hemodynamics and the functional status of some patients are already severely impaired at diagnosis, and attaining clinical stability and preventing progression is challenging. Variables measured during invasive right heart catheterization (RHC), imaging modalities, and laboratory or functional tests are used to assess the risk of deterioration and death [1,2,3], but non-invasive biomarkers that can repeatedly be assessed would be valuable for ongoing monitoring.

The nitric oxide (NO)–soluble guanylate cyclase (sGC)–cyclic guanosine monophosphate (cGMP) pathway is a major regulator of the pulmonary vascular tone [4], making it an attractive biomarker target given that endothelial dysfunction is central to PH pathophysiology. Importantly, altered endothelial NO synthase (eNOS) activity, decreased NO production, and dysregulated NO signaling in the pulmonary endothelium are known in different forms of PH [5,6,7]. Pulmonary NO production can be measured non-invasively in exhaled breath, potentially also reflecting the activity of the eNOS [8]. In patients with untreated PAH, studies measuring fractional exhaled NO concentrations at a 50 mL/s exhalation flow rate (*F*ENO_50_) have reported variable results [9,10,11], with unclear relationships to specific vasodilator therapy responses [10,11]. Notably, the acinar/alveolar concentration of NO, which can be calculated from exhaled NO concentrations measured at multiple flow rates, is described to be elevated in PAH in some studies [12,13], an observation that is not supported by other authors [11]. Hence, comprehensive data on exhaled NO parameters in a larger pool of patients with pre-capillary PH, both at diagnosis and after specific treatment, would add valuable clarification on the clinical utility of these biomarkers.

Currently, predictors used for risk stratification of patients with PAH include clinical factors, hemodynamic variables, measures of functional and exercise capacity, imaging markers of right heart function, and plasma N-terminal pro-brain natriuretic peptide (NT-proBNP) concentration [1]. On the contrary, exhaled NO measurements hold the potential to non-invasively monitor the underlying disease process at a molecular level and to directly assess the response to targeted therapy. Thus, we aimed to study whether alveolar and bronchial NO parameters can aid diagnosis and the prediction of therapeutic responses across pre-capillary PH subtypes, including groups 1, 3, and 4. In addition, we compared exhaled NO markers among the different groups of PH and examined associations with clinical parameters.

## 2. Materials and Methods

### 2.1. Study Design

Our single-center observational cohort study had a cross-sectional and a prospective part. We recruited patients with newly diagnosed pre-capillary PH and a relevant control group. In the prospective part, patients belonging to PH groups 1 and 4 were asked to attend a single follow-up visit during a routine clinical check-up in stable condition. The primary outcome was the difference in the alveolar NO concentration (*C*ANO) in patients at diagnosis compared to controls and the change in *C*ANO at the follow-up visit. Secondary outcomes included the difference in *F*ENO_50_ and *J*awNO between patients and controls and their temporal changes.

### 2.2. Enrolment of Subjects

**Figure 1:**
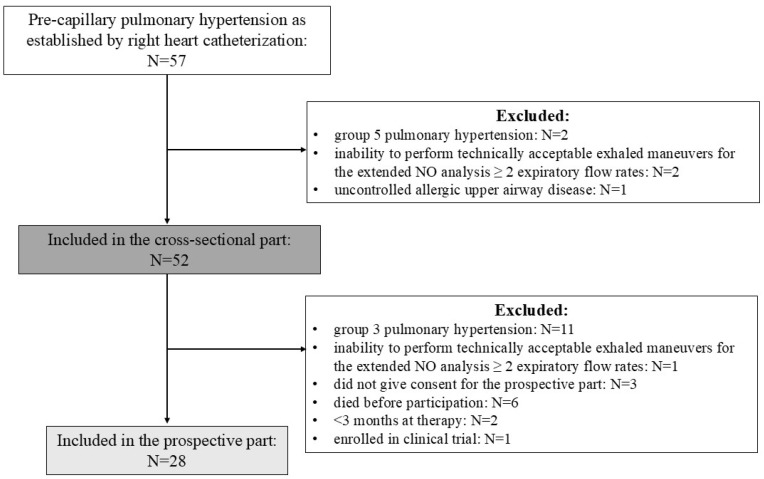
Flow chart of patient enrolment.

Inclusion criteria for patients were pre-capillary PH defined as mean pulmonary artery pressure (mPAP) > 20 mmHg, pulmonary artery wedge pressure (PAWP) ≤ 15 mmHg, and pulmonary vascular resistance (PVR) > 2 Wood units [1]. Only patients in PH groups 1, 3, and 4 were included; those belonging to PH group 5 were excluded. The recruitment period lasted from 1 September 2017 to 1 June 2025. Patients were included in the prospective part if they had group 1 or group 4 PH and were in stable condition as assessed by the treating physician, had unchanged pharmacological therapy for >3 months, or at least 3–6 months had elapsed after intervention due to chronic thromboembolic PH (CTEPH). The follow-up visit in patients with group 1 PH was conducted at ≥3 months of pulmonary vasodilator therapy and was planned at 6–12 months after the initiation of therapy when possible. Patients in a clinical trial at follow-up were excluded. Enrolment is shown in Figure 1.

Control subjects without chronic lung disease were volunteers among employees at the Department. Control subjects with chronic lung disease, either with chronic obstructive pulmonary disease (COPD) or interstitial lung disease (ILD), were included if the lung disease was in stable condition and there was no sign of PH on electrocardiogram or echocardiography [1]. ILD was established by the multidisciplinary ILD team at our center, and COPD was diagnosed according to the current guideline [14]. Control subjects were recruited between 1 January 2018 and 1 February 2019 and from 1 November 2024 to 1 June 2025.

Exclusion criteria for the whole study population, or reasons for a delayed follow-up, included fever or acute airway disease, e.g., rhinitis or bronchitis, 1 week prior to visits, uncontrolled allergic airway disease, chronic lung disease except for the control group with lung disease, and inability to perform technically acceptable exhalation maneuvers for NO analysis at at least 2 expiratory flow rates (100–250 mL/s).

During the outbreaks of the COVID-19 pandemic (from 1 March 2020 to 1 March 2022), recruitment of patients and control subjects significantly slowed down, and follow-up visits of patients were completed using telemedicine when possible or were often delayed. Exhaled NO measurements were not conducted in subjects with a suspected diagnosis of COVID-19 or within 2 weeks of the acute infection, and, in general, exhaled tests were avoided during the outbreaks to prevent potential viral spread among the healthcare staff. The uptake of COVID-19 vaccinations did not influence the timing of the exhaled NO measurements.

### 2.3. Clinical Assessment and Data Collection

All patients underwent a detailed clinical assessment to determine the clinical groups of PH following current guidelines [1]. Patients with group 1 PH received pulmonary vasodilator treatment and patients with CTEPH were treated with pulmonary endarterectomy, balloon pulmonary angioplasty, and/or pulmonary vasodilator therapy following the recommendations of the multidisciplinary expert panel at our center. At diagnosis, routine medical data, including demographics, parameters of RHC, echocardiography, plasma NT-proBNP concentration, pulmonary function test, diffusion capacity of the lung for carbon monoxide (D_LCO_), arterial blood gas analysis, 6 min walk distance (6MWD), and World Health Organization Functional Class (WHO-FC) [1], were recorded. At follow-up, we noted current therapy, 6MWD, WHO-FC, and plasma NT-proBNP concentration. Mortality risk was evaluated based on the three-strata and four-strata models at diagnosis and using the four-strata model at follow-up. Exhaled NO measurements were performed at both visits. More information on clinical measurements is available in the Supplementary Materials.

### 2.4. Exhaled Nitric Oxide Measurements

Subjects were not allowed to use inhaled medication, eat, drink, smoke, or perform pulmonary function tests or 6MWT one hour prior to measurements. Exhaled NO concentrations were measured at 50, 100, 150, 200, and 250 mL/s constant flow rates during an exhalation maneuver initiated from total lung capacity (Sievers Nitric Oxide Analyzer i280, GE Analytical Instruments, Boulder, CO, USA) [15,16]. Instrument calibration was carried out daily following the manufacturer’s instructions. The background ambient NO concentration was <5 ppb. Restrictors, as provided and calibrated by the manufacturer, were used to generate the required expiratory flows and ensure the closure of the velum. Maneuvers at different flows with a duration ≥ 6 s (50–100 mL/s) and ≥5 s (150–200–250 mL/s) were considered technically adequate [17]. NO readings were evaluated manually in a 3 s window with minimal sloping, with the exhalation flow being ±10% within the required rate after disregarding the initial expiratory volume of 150 mL air (i.e., anatomic dead space) [18]. The average value of two NO recordings (if available) at the same expiratory flow with <10% difference was calculated. NO concentrations at 100, 150, 200, and 250 mL/s exhalation flows were the inputs for the two-compartment model [17,19] to estimate *C*ANO and the total flux of NO in the conducting airway compartment (*J*awNO).

Eighty NO measurements were performed by patients (at diagnosis, N = 52; during follow-up, N = 28), and in 78 cases, NO output at all four exhalation flows was used in the model. Two patients (group 1 PH: N = 1, group 3 PH: N = 1) could not perform a valid maneuver at 250 mL/s flow rate at diagnosis, and in 12 cases, patients could perform only one adequate maneuver at certain flow rates (250 mL/s: N = 5, 200 mL/s: N = 3, 150 mL/s N = 2, 100 mL/s: N = 2). Therefore, no average NO value for these flow rates could be calculated, and a single exhaled NO value was used for the calculation of NO output. The mean r for the equation of the linear model was 0.96 (SD: 0.04). In 38 cases, r was <0.95 (48% of all measurements): r = 0.90–0.94: N = 14, r = 0.80–0.89: N = 24. All control subjects (N = 27) could perform 2 technically adequate maneuvers at all expiratory flow rates. In controls, the mean r was 0.96 (SD: 0.04) and r < 0.95 in 8 (30% of all cases): r = 0.90–0.94: N = 6, r = 0.80–0.89: N = 2. *F*ENO_50_ could not be obtained in three patients at diagnosis (1 patient in each PH group) and in one CTEPH patient at follow-up. All controls performed technically adequate exhalation maneuvers for the measurement of *F*ENO_50_.

### 2.5. Statistical Analysis

The primary outcome of the study was *C*ANO; therefore, based on our previous data in control subjects (group mean ± standard deviation: 2.3 ± 1.0 ppb) and patients with stable COPD (group mean ± standard deviation: 5.1 ± 3.6 ppb) [14], a priori sample size calculations were performed. The required sample sizes for the comparison of two independent groups were 15/group (effect size = 1.08, 1 − β = 0.80, α = 0.05). Continuous variables were compared with t-test, ANOVA with Bonferroni post-hoc test (mean ± standard deviation), and Wilcoxon, Mann–Whitney, and Kruskal–Wallis tests (median (interquartile range) in data with and without normal distributions, respectively. Spearman correlation was performed, and the receiver operating characteristic (ROC) curve was constructed to determine the best cut-off for *C*ANO to distinguish patients from controls (GraphPad Prism 10.4, GraphPad Software, San Diego, CA, USA). Cox regression was used to assess the link between exhaled NO parameters at diagnosis and survival of patients. To address whether the changes in *J*awNO are independently related to the change in 6MWD, multivariate linear regression analysis with backward stepwise selection was performed, removing variables with *p* > 0.20. Candidate variables included change in *J*awNO, PH group, age, sex, lung function parameters (FEV_1_% reference, FEV_1_/FVC, D_LCO_ % reference), baseline 6MWD, baseline 4-strata risk model, use of inhaled medications, and PH specific therapies at follow-up (phosphodiesterase-5 inhibitors (PDE5i), endothelin receptor antagonist (ERA), prostacyclin pathway activator (PPA)). Stata 18 software was used for regression analyses (StataCorp LLC, 2023, College Station, TX, USA).

## 3. Results

### 3.1. Characteristics of the Study Population

We recruited 52 patients with pre-capillary PH of different etiologies and a control group (Table 1, additional details are provided in the Supplementary Materials). Twenty-three patients had group 1 PH (idiopathic PAH: N = 19, PAH associated with connective tissue disease: N = 3, PAH associated with atrial septum defect: N = 1). Eleven patients were diagnosed with group 3 PH, while eighteen patients had group 4 PH (all CTEPH). Patients with group 3 PH had worse lung function, D_LCO_, 6MWD, and arterial blood gas parameters than patients with group 1 or group 4 PH.

RHC parameters were similar across PH groups; however, right ventricle-pulmonary artery uncoupling (tricuspid annular plane systolic excursion (TAPSE)/estimated systolic PAP ≤ 0.32) [1] was more often noted in PH groups 1 and 3, who also presented with worse WHO-FC than patients with group 4 PH (Table 2).

### 3.2. Exhaled NO Parameters at Diagnosis and Associations with Clinical Indicators

**Figure 2:**
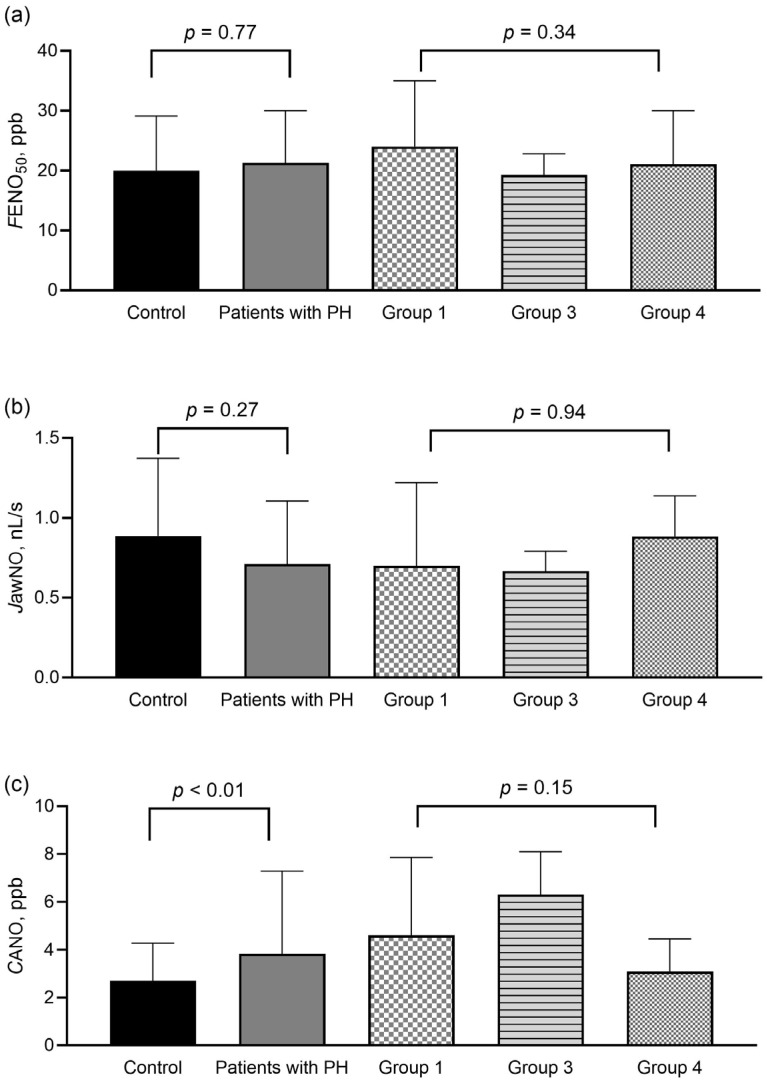
Exhaled nitric oxide parameters in control subjects and patients with precapillary pulmonary hypertension at diagnosis. *F*ENO_50_ (panel (**a**)), *J*awNO (panel (**b**)), and *C*ANO (panel (**c**)) were compared between control subjects (Mann–Whitney test) and among patients in different PH groups (Kruskal–Wallis test). Data are shown as median with upper interquartile range. *C*ANO: alveolar nitric oxide concentration; *F*ENO_50_: exhaled nitric oxide concentration measured at constant flow rates of 50 mL/s; *J*awNO: total flux of bronchial nitric oxide; PH: pulmonary hypertension; ppb: particles per billion.

We found no difference in *F*ENO_50_ and *J*awNO values between patients and control subjects or among patients with different PH etiologies (Figure 2a,b). However, *C*ANO was increased in PH (Figure 2c), and a cut-off > 2.82 ppb could separate patients from controls with a sensitivity of 73% and a specificity of 56% (area under the ROC curve = 0.68, *p* < 0.01). Moreover, a cut-off > 2.92 ppb could identify patients with group 1 and group 4 PH from controls without lung disease with a sensitivity of 66% and specificity of 74% (area under the ROC curve = 0.73, *p* < 0.01). No difference in *C*ANO was found among the clinical groups in PH (Figure 2c).

As COPD and ILD can alter *C*ANO [15,20], we also carried out an additional analysis, as shown in Supplementary Table S1. We compared clinical data between control subjects without chronic lung diseases and patients with group 1 or group 4 PH and between control subjects with lung diseases and patients with group 3 PH. We found no difference in clinical characteristics between control subjects and patients with group 1 or group 4 PH (*p* > 0.05); however, the *C*ANO was increased in group 1 PH (*p* = 0.002) and showed a trend of increase in group 4 PH (*p* = 0.08). Patients with group 3 PH had worse 6MWD and pulmonary function test values, but *C*ANO was not different compared to control subjects with chronic lung disease (*p* = 0.21). *F*ENO_50_ and *J*awNO were not different among patients and relevant control groups (*p* > 0.05).

**Figure 3:**
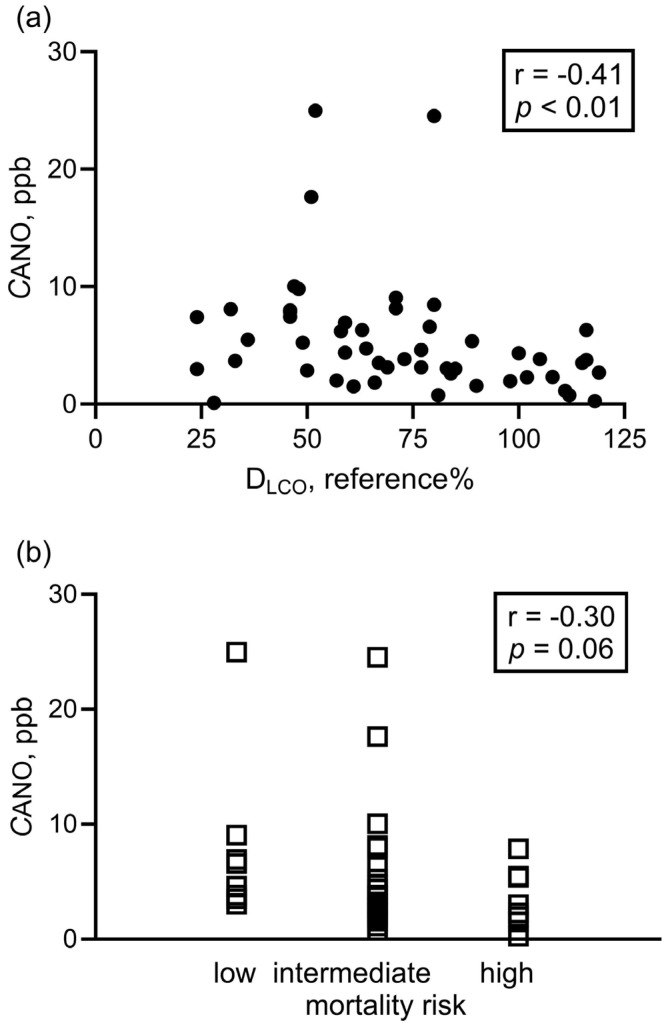
Correlation between alveolar nitric oxide concentration and clinical parameters at diagnosis. Spearman correlation was used to assess the correlation between *C*ANO and D_LCO_ (panel (**a**): all patients with PH, N = 50) and CANO vs. 3-strata risk assessment categories at diagnosis (panel (**b**): group 1 + group 4, N = 41). *C*ANO: alveolar nitric oxide concentration; D_LCO_: diffusion capacity of the lung for carbon monoxide.

D_LCO_ negatively correlated with *C*ANO in all patients (Figure 3a) and also in patients with group 1 and group 4 PH (*p* < 0.001, r = −0.52). Of note, there was a trend for association between *C*ANO and mortality risk category at diagnosis in PH groups 1 and 4 (Figure 3b). Other clinical parameters listed in Table 1 and Table 2 did not correlate with *C*ANO (*p* > 0.05).

During the observational period (median time: 24 months (11–57 months)), 14 patients died or received a lung transplantation (group 1: N = 4, group 3: N = 5, group 4: N = 5). Cox proportional hazard regression revealed no significant association between *F*ENO_50_, *J*awNO, or *C*ANO and transplant-free survival, either when all patients were analyzed together or separated by groups (hazard ratios did not differ from 1.0, *p* > 0.05).

### 3.3. Exhaled NO Parameters at Follow-Up

We measured exhaled NO parameters in 28 patients with groups 1 or 4 PH at a single follow-up visit. In PAH, the mean time difference between the two measurements was 13 months (min–max: 4–25 months). Patients received pulmonary vasodilator therapies in the whole timeframe between the two visits (at follow-up: PDE5i: N = 4; PDE5i + ERA: N = 6; PDE5i + ERA + PPA: N = 4; PDE5i + ERA + PPA + sotatercept: N = 1). In CTEPH, eight patients underwent interventions (pulmonary endarterectomy: N = 7; balloon pulmonary angioplasty, N = 1), out of whom one patient was receiving PDE5i treatment at follow-up. The average difference between the interventions and the follow-up was 20 months (min–max: 5–60 months). The remaining five patients with CTEPH did not undergo an intervention (two did not agree, three were not eligible) and were receiving oral pulmonary vasodilators at follow-up (PDE5i: N = 2, sGC stimulator: N = 3), which took place at an average of 26 months (min–max: 4–47 months) after the first measurement.

Importantly, the four-strata mortality risk score, the functional class, 6MWD, and the NT-proBNP levels improved at follow-up (Table 3).

**Figure 4:**
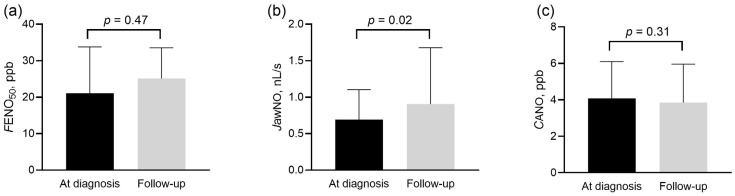
Exhaled nitric oxide parameters in patients with group 1 and 4 pulmonary hypertension at diagnosis and during follow-up. *F*ENO_50_ (panel (**a**)), *J*awNO (panel (**b**)), and *C*ANO (panel (**c**)) were compared at diagnosis and follow-up with the Wilcoxon sign rank test. *C*ANO: alveolar nitric oxide concentration; *F*ENO_50_: exhaled nitric oxide concentration measured at constant flow rates of 50 mL/s; *J*awNO: total flux of bronchial nitric oxide; ppb: particles per billion.

*J*awNO increased, but *C*ANO and *F*ENO_50_ remained unchanged at follow-up (Figure 4).

**Figure 5:**
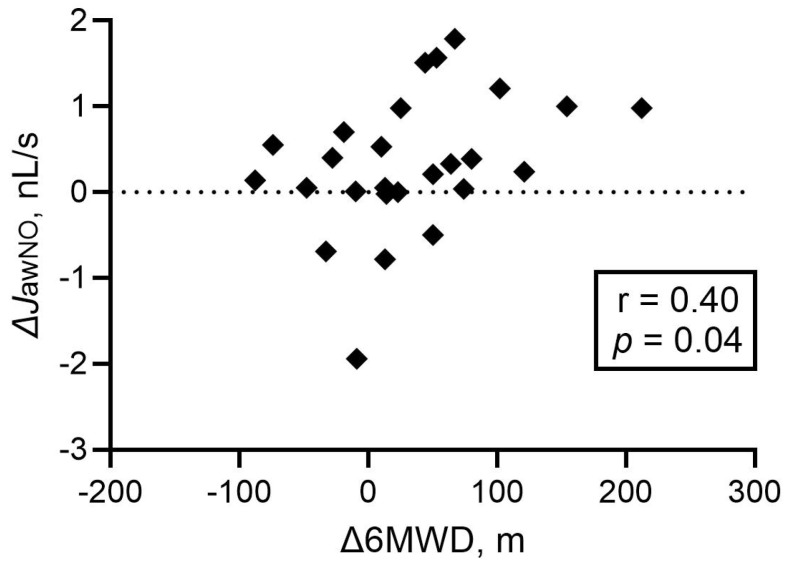
Correlation between the changes in *J*awNO and 6 min walk distance at follow-up. Spearman correlation was used. *J*awNO: total flux of bronchial nitric oxide; 6MWD: 6 min walk distance; Δ: difference between values at follow-up and diagnosis.

The increase in *J*awNO showed a positive correlation with the absolute increase in 6MWD (*p* = 0.04, r = 0.40, Figure 5) in the whole population and also in group 1 PH (*p* = 0.02, r = 0.59). The multivariate analysis confirmed that the change in *J*awNO was independently associated with the change in 6MWD, even after adjustment for demographic, lung function, and treatment-related variables (detailed results are shown in Supplementary Table S2). However, no relationship was noted to the change in NT-proBNP concentrations, WHO-FC, or the four-strata risk categories (*p* > 0.05).

## 4. Discussion

To our knowledge, this is the largest study to systematically register exhaled NO parameters of the central and peripheral airways in patients with pre-capillary PH and correlate them with important clinical variables, including survival and response to therapy. We used a previously validated method for exhaled NO measurements [15,16], and the linear model could be successfully used in all patients following international recommendations [17]. Our study revealed three main findings: (1) *C*ANO is increased in untreated pre-capillary PH patients, (2) *F*ENO_50_ and *J*awNO are unchanged at baseline compared to control subjects, and (3) *J*awNO increases following specific therapy initiation and correlates with functional improvement.

Bronchial and alveolar NO parameters are associated with the activities of different isoforms of pulmonary NOS. In non-smoking control volunteers, *J*awNO is influenced by both the constitutive (endothelial and neuronal NOS) and inducible NOS (iNOS), while *C*ANO is associated only with the constitutive isoforms [8]. However, the specific contribution of NOS isoforms to the exhaled NO parameters in PH is unknown.

We found that *C*ANO is increased in patients with untreated pre-capillary PH compared to all control subjects, although the reported cut-off value indicates a discriminative ability for this biomarker rather than diagnostic potential. Nonetheless, our data confirm previous findings in PAH [13,21] but also contradict another study, which reported unchanged *C*ANO compared to controls [11]. The net effect of the dysfunctions of various components of the NO signaling pathway, including eNOS activity, scavenging of the produced NO by superoxide, or arginase activity, leads to the decreased bioavailability of NO as a vasodilator molecule in the pulmonary vasculature in PH [22], and these processes can also influence alveolar NO production. To augment the abrogated NO signaling in the pulmonary vasculature, our results may indicate a compensatory upregulation of NO synthesis in acinar/alveolar regions, potentially including respiratory epithelial cells via eNOS [23] or nNOS [24], while under hypoxic conditions, iNOS activity could also contribute [25]. These mechanisms remain hypothetical and warrant further investigation. Although *C*ANO was not related to hemodynamic, functional, or laboratory parameters, we observed a trend for a link between decreased *C*ANO and higher mortality risk in patients with PAH and CTEPH at diagnosis, which should be re-assessed in a larger population of PH groups. In addition, we did not find a difference in *C*ANO between control subjects with ILD/COPD and patients with PH due to lung diseases, as also shown for patients with systemic sclerosis with and without PAH [21], which is consistent with the hypothesis that increased *C*ANO may reflect alveolar/acinar inflammation due to iNOS activity.

We also demonstrated that an increased *C*ANO in pre-capillary PH is moderately associated with a compromised diffusion of the lungs for carbon monoxide, which was previously also shown in bronchial asthma [16], systemic sclerosis-ILD without PH [21], and pulmonary sarcoidosis [26]. Although our data did not allow us to explore a causal relationship, the altered transfer via the alveolocapillary membrane can also result in a reduced fraction of the alveolar NO production reaching the pulmonary capillaries. Furthermore, decreased pulmonary capillary circulation in all forms of PH and the ventilation/perfusion mismatch present in CTEPH and COPD may limit the availability of hemoglobin in red blood cells in the pulmonary circulation to absorb NO. These processes, besides the possibility of increased alveolar NO production, may also influence *C*ANO and result in an altered steady-state concentration.

The complex processes behind the regulation of *C*ANO may also explain why we did not find a change in this biomarker following therapy when a clear clinical improvement was observed in patients with group 1 and 4 PH. Although the compensatory upregulation of alveolar NO production may be present, the presumably increased cardiac output and the improved pulmonary circulation could absorb more NO, which would leave the *C*ANO unchanged. Similarly, no change in *C*ANO was reported in PAH after 3-month therapy with an endothelin receptor antagonist [11].

Bronchial NO parameters (*F*ENO_50_ and *J*awNO) were not different in patients, even if different clinical groups of PH were analyzed separately. This corroborates previous results describing that *F*ENO_50_ is not a biomarker for idiopathic PAH [10] or PH associated with ILD [27] but contradicts the findings by others in PAH and CTEPH [28]. Similarly, some authors reported a change [12,29] while others did not find a change [10,28] in *F*ENO_50_ after pulmonary vasodilator treatment. Besides the low sample sizes of the previous studies (between 10 and 31), this discrepancy might also be explained by the individual variability of pulmonary vascular remodeling or the compensatory increase in bronchial blood flow as a pool for NO absorption.

Unexpectedly, we found that *J*awNO was increased at follow-up in patients with group 1 and 4 PH when adequate therapy was initiated or the patients underwent interventions. Of note, the magnitude of change in *J*awNO was directly proportional to the increase in 6MWD, an important parameter during the clinical assessment, and this association was independent of demographic, lung function, and treatment-related variables. This relation was even more pronounced when patients with group 1 PH were analyzed separately. *J*awNO depends on the airway tissue concentration of NO [19]. All patients with group 1 PH and 5 out of the 13 patients with group 4 PH were receiving a drug targeting the NO-sGC-cGMP pathway. Indeed, it has been demonstrated in vascular smooth muscle cells that cGMP upregulates iNOS gene expression and increases NO production [30]. Apart from the pulmonary vasculature, PDE5 is also found in human alveolar epithelial cells [31]. Hence, the increase in *J*awNO can implicate the effectiveness of the cGMP accumulation in bronchial epithelial cells (and possibly also in the pulmonary arterial smooth muscle cells) and the consequential induction of iNOS, which results in increased airway wall NO concentration.

The non-invasive assessment of pulmonary NO dynamics in the exhaled breath offers an attractive clinical tool. Importantly, patients did not report any adverse events; however, some patients experienced coughing during the exhalation maneuvers and fatigue that demanded a pre-term finish of the measurement session and fewer exhalation maneuvers than planned. Still, our data suggest that the monitoring of *J*awNO might indicate a positive response at the cellular level to drugs targeting the NO pathway and could be used as a biomarker even when clinical response is less evident or delayed in clinical settings.

Future studies shall focus on more deeply exploring the connection between the change in exhaled NO parameters and the improvement in pulmonary hemodynamics as assessed by repeated right heart catheterizations. Furthermore, exhaled NO parameters shall consecutively be monitored at each follow-up visit to give a better insight into the association between pulmonary NO dynamics and disease control and to determine the clinically relevant temporal changes in *J*awNO.

Our study has strengths and limitations. Group 1 and 4 PH forms are rare diseases, and recruitment and follow-up were challenged by the pandemic. Nonetheless, we recruited a relatively high number of patients. In the prospective part, patients attended only a single follow-up visit, planned 6–12 months after the initiation of pulmonary vasodilator therapy or intervention. Thus, the interpretation of data at follow-up is limited by the heterogeneity of the group with respect to treatment modalities and the absence of RHC data to directly assess the improvement in pulmonary hemodynamics and relate it to the change in exhaled NO parameters. Of note, we fully followed the recommendations of the current technical standard document to measure exhaled NO concentration and use the linear method for the extended NO analysis [17] and also conformed to the instrument’s instructions set by the manufacturer; however, 48% of all measurements in patients produced a regression line with an r-value less than the limit of reliability, i.e., r < 0.95 [17]. In our view, in this patient population, this can be attributed to cough induced by the enlarged pulmonary arteries and the swings in intrathoracic pressure during the multiple exhalation maneuvers. Future studies should take this limitation into account when using this measurement protocol in patients with PH and focus on exploring modifiable clinical and technical factors to improve measurement reliability.

## 5. Conclusions

Alveolar NO concentration is elevated in pre-capillary PH, suggesting upregulated NO production in the peripheral airways. *J*awNO is associated with the improvement in exercise capacity in patients with PAH and CTEPH after adequate therapy and may reflect the improved pulmonary circulation and a better response to pulmonary vasodilator therapy and interventions. We have provided evidence that exhaled NO parameters calculated using the two-compartment model can aid a better understanding of pulmonary NO signaling in pre-capillary PH and may serve as novel biomarkers.

## Figures and Tables

**Table 1:** Clinical characteristics of subjects.

	Control Subjects	Patients with Pre-Capillary PH	*p*-Value	Patients with Pre-Capillary PH			*p*-Value
				Group 1	Group 3	Group 4	
N	27	52		23	11	18	
Male, N (%)	12 (44)	26 (50)	0.81	10 (44%)	9 (82%)	7 (39%)	0.06
Age, years	54 ± 10	58 ± 15	0.27	55 ± 15	65 ± 11	56 ± 16	0.18
Never/former/current smoker, N	15/10/2	40/11/1	0.12	18/4/1	6/5/0	16/2/0	0.16
Pack-years	26 (13–47)	30 (20–40)	0.60	30 (15–32)	40 (30–63)	17 (10–21)	0.07
Body mass index, kg/m^2^	28.0 ± 6.0	29.0 ± 6.9	0.50	28.8 ± 6.2	27.0 ± 6.4	30.8 ± 7.8	0.28
Inhaled therapy, N (%)			NA				NA
Inhaled corticosteroid	0	7 (13)		2 (9)	3 (27)	2 (11)	
Long-acting beta_2_-agonist	2 (7)	8 (15)		3 (13)	3 (27)	1 (6)	
Long-acting anticholinergic	4 (15)	6 (12)		2 (9)	3 (27)	0	
Systemic corticosteroid use, N (%)	0	6 (12)	0.09	0	5 (45)	1 (6)	**<0.001**
Respiratory comorbidities, N (%)			0.92				**<0.001**
Bronchial asthma	1 (4)	3 (6)		1 (4)	0	2 (11)	
COPD	4 (15)	6 (12)		2 (9)	4 (36)	0	
ILD	4 (15)	7 (13)		0	7 (64)	0	
OSA	0	1 (2)		0	1 (9)	0	
FVC, L	3.60 ± 0.74	3.23 ± 1.26	0.17	3.22 ± 1.11	2.38 ± 0.77 ^##^	3.77 ± 1.40	**0.01**
FVC, % reference	96 ± 13	92 ± 14	0.06	91 ± 21	64 ± 20 ** ^###^	97 ± 19	**<0.001**
FEV_1_, L	2.77 ± 0.63	2.33 ± 0.93	0.10	2.45 ± 0.87	1.78 ± 0.49 ^##^	2.83 ± 1.00	**<0.01**
FEV_1_, % reference	90 ± 16	82 ± 22	0.09	84 ± 17	61 ± 20 ** ^###^	93 ± 21	**<0.001**
FEV_1_/FVC	0.78 ± 0.13	0.76 ± 0.10	0.66	0.76 ± 0.10	0.78 ± 0.13	0.76 ± 0.08	0.92
D_LCO_, % reference	87 ± 19 ^N = 8^	72 ± 2	NA	77 ± 30	47 ± 19 ** ^#^	82 ± 16	**<0.01**
6MWD, m	465 ± 76 ^N = 8^	350 ± 137	NA	366 ± 125	252 ± 101 ^#^	388 ± 148	**0.02**

Data are shown as mean ± SD or median (interquartile range) and analyzed with *t*-test or Mann–Whitney test, ANOVA, or Kruskal–Wallis test with post-hoc tests, Fisher’s exact, or chi-square tests. Significant differences are highlighted in bold. ** *p* < 0.01 vs. PAH, ^#^ *p* < 0.05 ^##^ *p* < 0.01 ^###^ *p* < 0.01 vs. Group 4. D_LCO_, 6MWD were only measured in controls with chronic lung diseases, and therefore were not compared with values in patients with PH. COPD: chronic obstructive pulmonary disease; D_LCO_: diffusion capacity of the lung for carbon monoxide; FEV_1_: forced expiratory volume in 1 s; FVC: forced vital capacity; ILD: interstitial lung disease; L: liter; N: number; NA: not applicable due to small sample size; OSA: obstructive sleep apnea; PH: pulmonary hypertension; 6MWD: 6 min walk distance.

**Table 2:** Right heart catheterization, echocardiographic, functional, and laboratory data of patients with pre-capillary pulmonary hypertension.

	Total N = 52	Group 1 N = 23	Group 3 N = 11	Group 4 N = 18	*p*-Value
mPAP, mmHg	46 (37–54)	52 (39–59)	44 (36–49)	44 (37–52)	0.21
PVR, Wood Unit	8.5 (5.8–12.5)	9.8 (5.9–14.7)	9.8 (5.7–12.1)	7.6 (4.6–10.0)	0.22
PAWP, mmHg	8 (6–11)	8 (7–11)	8 (5–10)	10 (6–14)	0.57
Right atrial pressure, mmHg	10 (4–15)	9 (3–13)	6 (2–13)	14 (6–19)	0.11
Cardiac index, L/min/m^2^	2.1 (1.9–2.7)	1.9 (1.7–2.8)	2.2 (2.0–2.7)	2.1 (1.9–2.7)	0.58
Stroke volume index, mL/m^2^	28 (23–36)	28 (21–37)	28 (22–36)	27 (24–33)	0.70
SvO_2_, %	66 (62–69) ^N = 42^	66 (61–70) ^N = 21^	67 (63–69) ^N = 9^	65 (62–68) ^N = 12^	0.86
TAPSE, mm	17 (13–20)	14 (11–20)	18 (13–19)	20 (16–22)	0.13
Estimated sPAP, mmHg	74 (59–93)	86 (65–94)	79 (63–96)	62 (41–89)	0.07
TAPSE/estimated sPAP, mm/mmHg	0.22 (0.14–0.34)	0.16 (0.12–0.29)	0.20 (0.14–0.29)	0.32 (0.22–0.49) *	**0.02**
RV-PA uncoupling, N (%)	37 (71)	18 (78)	10 (91)	9 (50)	**0.04**
Right atrial area, cm^2^	25 (18–31)	26 (21–31)	26 (21–32)	21 (17–30)	0.46
WHO functional classes, N (%)					**0.01**
I or II	10 (19)	2 (9)	0	8 (44)	
III	38 (73)	20 (87)	10 (91)	8 (44)	
IV	4 (8)	1 (4)	1 (9)	2 (12)	
NT-proBNP, pg/mL	1676 (433–3297)	2161 (455–3461)	2202 (645–4107)	785 (169–2778)	0.28

Data are shown as a median (interquartile range) or number (percentage), and clinical groups of pulmonary hypertension were compared with the Kruskal–Wallis test with post-hoc analysis or chi-square test. Significant differences are highlighted in bold. * *p* < 0.05 vs. Group 1. mPAP: mean pulmonary arterial pressure; N: number; NT-proBNP: N-terminal pro-brain natriuretic peptide; PVR: pulmonary vascular resistance; RV-PA: right ventricle-pulmonary artery; sPAP: systolic pulmonary arterial pressure; SvO_2_: mixed venous oxygen saturation; TAPSE: tricuspid annular plane systolic excursion; WHO: World Health Organization.

**Table 3:** Patient characteristics at diagnosis and during follow-up (N = 28).

	Diagnosis	Follow-Up	*p*-Value
WHO functional classes, N			**<0.001**
I or II	10	21	
III	16	7	
IV	2	0	
6MWD, m ^N = 26^	403 ± 123	435 ± 123	**0.02**
NT-proBNP, pg/mL	881 (282–2667)	517 (220–1636)	**0.03**
Four-strata risk category			**<0.01**
Low	5	10	
Intermediate low risk	9	13	
Intermediate high risk	13	2	
High risk	1	3	

Data are shown as mean ± standard deviation or median (interquartile range) and analyzed with paired *t*-test, Wilcoxon sign rank, or chi-square tests. Significant differences are highlighted in bold. NT-proBNP: N-terminal pro-brain natriuretic peptide; WHO: World Health Organization; 6MWD: 6 min walk distance.

## Data Availability

The data that support the findings of this study are available from the corresponding author upon reasonable request.

## Supplementary Materials

The following supporting information can be downloaded at: https://www.mdpi.com/article/10.3390/biomedicines13122957/s1, Table S1: Comparison of clinical data between patients and controls with and without lung diseases. Table S2: Multivariate regression analysis on predictors for change in 6-minute walk distance. Whole model *p* = 0.001, R^2^ = 0.77. References [32,33,34] appear the Supplementary Materials.

## Abbreviations

The following abbreviations are used in this manuscript:

NO	Nitric oxide
PH	Pulmonary hypertension
*C*ANO	Alveolar nitric oxide concentration
*J*awNO	Total bronchial flux of nitric oxide
PAH	Pulmonary arterial hypertension
RHC	Right heart catheterization
sGC	Soluble guanylate cyclase
cGMP	Cyclic guanosine monophosphate
eNOS	Endothelial NO synthase
*F*ENO_50_	Fractional exhaled NO concentrations at 50 mL/s exhalation flow rate
mPAP	Mean pulmonary artery pressure
PAWP	Pulmonary artery wedge pressure
PVR	Pulmonary vascular resistance
CTEPH	Chronic thromboembolic pulmonary hypertension
COPD	Chronic obstructive pulmonary disease
ILD	Interstitial lung disease
NT-proBNP	N-terminal pro-brain natriuretic peptide
D_LCO_	Diffusion capacity of the lung for carbon monoxide
6MWD	6 min walk distance
WHO-FC	World Health Organization Functional Class
ROC	Receiver operating characteristic
FEV_1_	Forced expiratory volume in 1 s
FVC	Forced vital capacity
L	Liter
N	Number
NA	Not applicable
OSA	Obstructive sleep apnea
TAPSE	Tricuspid annular plane systolic excursion
RV-PA	Right ventricle-pulmonary artery
sPAP	Systolic pulmonary arterial pressure
SvO_2_	Mixed venous oxygen saturation
ppb	Particles per billion
PDE5i	Phosphodiesterase-5 inhibitor
ERA	Endothelin receptor antagonist
Δ	Difference between values at follow-up and diagnosis
iNOS	Inducible nitric oxide synthase
nNOS	Neuronal nitric oxide synthase
