# Supplementary material for: Alveolar and Bronchial Nitric Oxide Parameters in Pre-Capillary Pulmonary Hypertension
Source: Biomedicines. 2025 Dec 1;13(12):2957. doi: 10.3390/biomedicines13122957 (PMC12730688; doi:10.3390/biomedicines13122957)
Supplement: Supplementary file 1 [file biomedicines-13-02957-s001.zip › biomedicines-3985282-supplementary.pdf]

Alveolar and bronchial nitric oxide parameters in pre-capillary pulmonary hypertension

Supplementary document

Recording and measurements of routine parameters

All patients underwent detailed clinical assessment including the evaluation of medical history, echocardiography, arterial blood gas analysis, pulmonary angio-CT, pulmonary ventilation/perfusion scan (in suspicion of pulmonary thromboembolic disease), pulmonary function testing, 6-minute walk test, blood tests (including immune serology when needed), determination of World Health Organization Functional Class (WHO-FC). Clinical groups of PH were determined based on these assessments following current guidelines[1]. All patients with group 1 PH received pulmonary vasodilator treatment and patients with CTEPH were treated with pulmonary endarterectomy (PEA), balloon pulmonary angioplasty (BPA) and/or pulmonary vasodilator therapy following the recommendations of the multidisciplinary expert panel at our center. Data were recorded in the routine medical documents, only exhaled NO measurements were performed as an additional examination. At diagnosis, we collected demographic data (age, sex, body mass index (BMI)), comorbidities, regular medical therapy, parameters of the RHC examination (right atrial pressure (RAP), mean/systolic/diastolic pulmonary arterial pressure (PAP), pulmonary vascular resistance (PVR), pulmonary arterial wedge pressure (PAWP), cardiac index (CI), stroke volume index (SVI), , mixed venous oxygen saturation (SvO_2)) echocardiographic data (tricuspid annular plane systolic excursion (TAPSE), estimated systolic PAP), plasma N-terminal pro-brain natriuretic peptide (NT-proBNP) concentration, pulmonary function test results (diffusion capacity of the lung for carbon monoxide (D_{LCO}), forced expiratory volume in 1 second (FEV_1), forced vital capacity (FVC)), arterial blood gas values (pH, pO_2, pCO_2), 6-minute walk distance (6MWD) and WHO-FC. At follow-up, we recorded current therapy, 6MWD, WHO-FC, plasma NT-proBNP concentration. In addition, mortality risk was evaluated based on the three-strata and four strata models at diagnosis and using the four-strata model at follow-up.

In control subjects, data on prior medical history, comorbidities, regular therapy were collected, and patients underwent spirometry and exhaled NO measurements. In control subjects with lung disease, we also measured diffusion capacity of the lung for carbon monoxide (D_{LCO}). RHC was performed with Corodyn 7F catheters (B Braun SE, Melsungen, Germany) under standard conditions[1] as previously described[2]. We measured RAP, /diastolic/mean/systolic PAP and PAWP. Cardiac output (CO) was determined by the thermodilution method, and SvO_2 was measured from a pulmonary artery blood sample. Vasoreactivity test was performed with inhaled iloprost, no patients showed vasoreactivity. Further parameters were calculated as CI=CO/body surface area, SVI=CO/heart rate/body surface area, PVR=(mean PAP-PAWP)/CO). Echocardiography was performed with the Mindray DC-70 X-Insight instrument (Shenzhen Mindray Bio-Medical Electronics Co., Shenzen, China) as previously described[2]. Right ventricle-pulmonary artery coupling was determined (TAPSE/estimated systolic PAP > 0.32)[1]. Plasma NT-proBNP concentration, was measured in the Central Laboratory of the Clinical Centre at Semmelweis University. Arterial blood gas values and SvO_2 were determined with a blood gas analyser (Roche Cobas b 221, Basel, Switzerland). 6MWT was performed along a 20-m long corridor, with continuous monitoring of oxygen saturation and heart rate in addition to blood pressure measurement and evaluation of the BORG 0-10 dyspnoea scale before and after the test. Pulmonary function tests were performed according to international guidelines[3,4] (PDT-111 spirometer and PPF-18 D_{LCO} device, Piston, Budapest Hungary).

Clinical characteristics and exhaled NO data in subgroups of controls and patients with PH

We also compared clinical data between controls without chronic lung diseases and patients with group 1 or group 4 PH, and also between controls with lung diseases and patients with group 3 PH (Table S1). We found no difference in clinical characteristics between control

subjects and patients group 1 or group 4 PH ($p>0.05$), however, the C_{ANO} was increased in group 1 PH and showed a trend for an increase in group 4 PH. Patients with group 3 PH had worse 6MWD, and pulmonary function test values, but C_{ANO} was not different compared to control subjects with chronic lung disease ($p=0.21$). $F_{ENO_{50}}$ and J_{awNO} were not different among patients and relevant control groups ($p>0.05$).

Multivariate regression analysis on predictors for change in 6-minute walk distance

To address potential predictors of the change in 6MWD at follow-up from baseline, we performed a multivariate linear regression analysis with backward stepwise selection, removing variables with $p>0.20$. Candidate variables included change in J_{awNO}, PH group, age, sex, lung function parameters at baseline (FEV_1 % reference, FEV_1/FVC, D_{LCO} % reference), 6MWD at baseline, 4-strata risk model at baseline, use of inhaled medications at baseline (ICS, LABA, LAMA), and PH specific therapies at follow-up (PDE5 inhibitors, endothelin receptor antagonists, prostacyclin pathway activators). In the final model, the change in J_{awNO}, use of LAMA, use of prostacyclin pathway activators, FEV_1/FVC ratio, D_{LCO} % reference, and baseline 6MWD were each independently associated with change in 6MWD, with change in J_{awNO} remaining the strongest independent predictor even after adjustment for demographic, lung function, and treatment related variables (whole model $p=0.001$, $R^2=0.77$, Table S2). This supports our conclusion that J_{awNO} is independently associated with functional improvement in patients with PAH and CTEPH at follow-up. Stata 18 software was used for regression analyses (StataCorp LLC, 2023, College Station, TX, USA).

References

1. Humbert, M.; Kovacs, G.; Hoeper, M.M.; Badagliacca, R.; Berger, R.M.F.; Brida, M.; Carlsen, J.; Coats, A.J.S.; Escribano-Subias, P.; Ferrari, P.; et al. 2022 ESC/ERS Guidelines for the Diagnosis and Treatment of Pulmonary Hypertension. *Eur Heart J* **2022**, *43*, 3618–3731, doi:10.1093/eurheartj/ehac237.
2. Csósza, G.; Valkó, L.; Dinya, E.; Losonczy, G.; Müller, V.; Lázár, Z.; Karlócai, K. Right Ventricular Stroke Work Index in Pulmonary Arterial Hypertension and Chronic Thromboembolic Pulmonary Hypertension: A Retrospective Observational Study. *Pulm Circ* **2024**, *14*, e12433, doi:10.1002/pul2.12433.
3. Graham, B.L.; Steenbruggen, I.; Miller, M.R.; Barjaktarevic, I.Z.; Cooper, B.G.; Hall, G.L.; Hallstrand, T.S.; Kaminsky, D.A.; McCarthy, K.; McCormack, M.C.; et al. Standardization of Spirometry 2019 Update. An Official American Thoracic Society and European Respiratory Society Technical Statement. *Am J Respir Crit Care Med* **2019**, *200*, e70–e88, doi:10.1164/rccm.201908-1590ST.
4. Graham, B.L.; Brusasco, V.; Burgos, F.; Cooper, B.G.; Jensen, R.; Kendrick, A.; MacIntyre, N.R.; Thompson, B.R.; Wanger, J. 2017 ERS/ATS Standards for Single-Breath Carbon Monoxide Uptake in the Lung. *European Respiratory Journal* **2017**, *49*, doi:10.1183/13993003.00016-2016.

Table S1. Comparison of clinical data between patients and controls with and without lung diseases					
	Control subjects N=27		Patients with pre-capillary pulmonary hypertension N=52		
	Without chronic lung disease	With chronic lung disease	Group1	Group 3	Group 4
N	19	8	23	11	18
Male, N (%)	6 (32%)	6 (75%)	10 (44%)	9 (82%)	7 (39%)
Age, years	51 ± 8	65 ± 11	55 ± 15	65 ± 11	56 ± 16
Never/former/current smoker, N	14/5/0	1/5/2	18/4/1	6/5/0	16/2/0
Pack-years	20 (10-25)	30 (20-80)	30 (15-32)	40 (30-63)	17 (10-21)
Body mass index, kg/m^2	27.5 ± 6.7	28.0 ± 6.0	28.8 ± 6.2	27.0 ± 6.4	30.8 ± 7.8
Inhaled therapy, N*					
ICS	0	0	1 (4%)	3 (27%)	2 (11%)
LABA	0	2 (25%)	2 (8%)	4 (36%)	0
LAMA	0	4 (50%)	2 (8%)	4 (36%)	0
**Systemic corticosteroid, N**	0	0	0	**5 (45%)$^{\#}$**	1 (6%)
Presence of respiratory diseases					
Bronchial asthma, N	1	0	1	0	2
COPD, N	0	4	2	4	0
ILD, N	0	4	0	7	0
OSA, N	0	0	0	1	0
**FVC, L**	3.67 ± 0.74	3.44 ± 0.75	3.22 ± 1.11	**2.38 ± 0.77$^{\#\#}$**	3.77 ± 1.40
**FVC, % reference**	98 ± 12	92 ± 14	91 ± 21	**64 ± 20$^{\#\#}$**	97 ± 19
**FEV_1, L**	2.96 ± 0.55	**2.33 ± 0.63***	2.45 ± 0.87	**1.78 ± 0.49$^{\#}$**	2.83 ± 1.00
**FEV_1, % reference**	97 ± 10	**74 ± 16*****	84 ± 17	**61 ± 20**	93 ± 21
**FEV_1/FVC**	0.81 ± 0.03	**0.70 ± 0.22***	0.76 ± 0.10	**0.78 ± 0.13**	0.76 ± 0.08
D_{LCO}, % reference	Not measured	87 ± 19	77 ± 30	**47 ± 19 $^{\#\#}$**	82 ± 16
6MWD, m	Not measured	465 ± 76	366 ± 125	**252 ± 101$^{\#\#\#}$**	388 ± 148
Arterial blood gas	Not measured				
pH		7.41 ± 0.02	7.42 ± 0.03	**7.38 ± 0.03$^{\#}$**	7.43 ± 0.04

pO_2, mmHg<br>pCO_2, mmHg		62 ± 13<br>41 ± 9	65 ± 15<br>32 ± 6	54 ± 8<br>42 ± 9	65 ± 10<br>32 ± 4
*F*ENO50, ppb	29 (20-42)	43 (16-58)	35 (24-81)	23 (19-40)	30 (21-44)
***C*ANO, ppb**	2.27 (1.80-3.24)	4.36 (3.31-5.13)	**4.61 (2.67-7.86)****	6.31 (2.99-8.10)	**3.10 (2.18-4.64)&**
*J*awNO, nL/s	1.03 (0.49-1.20)	0.81 (0.18-2.32)	0.70 (0.39-1.22)	0.67 (0.58-0.79)	0.88 (0.24-1.13)

Data are shown as mean ± SD or median (interquartile range) and analyzed with t-test or Mann-Whitney test, or Fisher exact. Bold letters indicate the parameter with significant difference among the groups. &p=0.08, *p<0.05 **p<0.01, ***p<0.001 vs. control without chronic lung disease, #p<0.05 ##p<0.01 ###p<0.001 vs. control with chronic lung disease. DLCO, 6MWD and arterial blood gas parameters were only measured in control subjects in the subgroup with chronic lung disease. *C*ANO: alveolar nitric oxide concentration, COPD: chronic obstructive pulmonary disease, DLCO: diffusion capacity of the lung for carbon monoxide, *F*ENO50: exhaled nitric oxide concentration measured at constant flow rates of 50 ml/s, FEV_1: forced expiratory volume in 1 second, FVC: forced vital capacity, ICS: inhaled corticosteroid, ILD: interstitial lung disease, *J*awNO: total flux of bronchial nitric oxide, L: Litre, LABA: long-acting beta2 agonist, LAMA: long-acting muscarinic antagonist, N: number, NA: not applicable due to small sample size, OSA: obstructive sleep apnoea, 6MWD: 6-minute walk distance.

**Table S2. Multivariate regression analysis on predictors for change in 6-minute walk distance. Whole model p=0.001, R^2=0.77.**				
	Coefficient	P-value	95% confidence interval	
**Change in JawNO**	**53.25902**	**<0.001**	**29.3441**	**77.17395**
PH group	-15.99344	0.100	-35.4035	3.416606
**Treatment with long-acting antimuscarinic drugs**	**-129.6111**	**0.039**	**-251.8433**	**-7.378966**
Female sex	27.97508	0.156	-11.86304	67.81319
**Treatment with prostacyclin pathway activators**	**72.53197**	**0.049**	**0.3895691**	**144.6744**
**FEV_1/FVC**	**-344.0727**	**0.019**	**-622.9825**	**-65.163**
**D_{LCO} % reference**	**1.252236**	**0.018**	**0.2451098**	**2.259363**
**Baseline 6-minute walk distance**	**-0.3506827**	**0.001**	**-0.5356232**	**-0.1657422**
Treatment with phosphodiesterase-5 inhibitors	-49.18057	0.133	-115.0586	16.69744
D_{LCO}: diffusion capacity of the lung for carbon monoxide, FEV_1: forced expiratory volume in 1 second, FVC: forced vital capacity, *J*awNO: total flux of bronchial nitric oxide.				
